# Association between Prepartum Feeding Behavior and Periparturient Health Disorders in Dairy Cows

**DOI:** 10.3389/fvets.2016.00065

**Published:** 2016-08-22

**Authors:** Karen M. Luchterhand, Paula R. B. Silva, Ricardo C. Chebel, Marcia I. Endres

**Affiliations:** ^1^Department of Animal Science, University of Minnesota, St. Paul, MN, USA; ^2^Department of Veterinary Population Medicine, University of Florida, Gainesville, FL, USA; ^3^Department of Large Animal Clinical Sciences, University of Florida, Gainesville, FL, USA

**Keywords:** prepartum behavior, feeding time, periparturient cow health, cow behavior

## Abstract

The objective of this study was to investigate the relationship between prepartum feeding behavior, measured as time spent feeding per day, and periparturient health disorders, milk yield, milk composition, and milk somatic cell count in Jersey cows. Pregnant Jersey cows were marked with unique alphanumeric symbols and were moved into a prepartum group 4 weeks prior to their expected calving date. At enrollment, cows with a body condition score <2 or >4 or a locomotion score >3 were not included. Time spent feeding was measured using 10-min video scan sampling for 24-h periods of 2–4 days per week of the study. A total of 925 cows were eligible for analysis. Parity was based on lactation number at the time of enrollment and classified as nulliparous (cows pregnant with their first calf), primiparous (cows pregnant with their second calf), and multiparous (lactation ≥2). Multiparous cows with two or more health disorders spent approximately 10% less time feeding prepartum than cows that did not have any health disorders. Multiparous cows subsequently diagnosed with metritis had a tendency to spend 5% less time feeding prepartum than healthy counterparts. Primiparous cows with retained placenta had a 10% reduction in feeding time compared to healthy primiparous cows. Monitoring time spent feeding prepartum by primiparous and multiparous cows, even on a limited number of days, appeared to be beneficial in predicting cows at risk for periparturient health disorders. Real-time daily feeding behavior monitoring technologies that can be used by dairy farms are now available, which might prove to be even more helpful in identifying cows at risk for periparturient cow health disorders as more data points can be recorded for each cow and compared to her own behavior or that of specific cohorts.

## Introduction

Early detection of health disorders would potentially prevent severity or reduce the duration of health problems and reduce on-farm mortality. The periparturient (or transition) period usually defined as 3 weeks before and 3 weeks after calving (1) is considered one of the most critical periods of a dairy cow’s life, with the largest number of health disorders happening in the first 10 days in milk (2). During the periparturient period, cows are at risk for metabolic and infectious disorders, which can ultimately affect reproductive performance (3). It has been estimated that approximately 50% of the cows have one or more adverse health events during this period (4). Reducing morbidity and mortality can improve animal welfare and farm profitability by reducing treatment costs, preventing reduction of milk yield, improving reproductive performance, and minimizing premature culling or death (5).

Changes in feeding behavior may become particularly useful for early detection of health disorders with the increased use of automated technologies occurring on dairy farms. Some monitoring systems have been validated and the data generated by them are highly correlated to direct observation (6). Previous research with dairy cattle has indicated cows that had a case of metritis had reductions in time spent feeding (7) and dry matter intake (DMI) (8) during the periparturient period compared with healthy cows. Cows diagnosed with ketosis had rapid decreases in time spent feeding, feed intake, and feeding rate approximately 4 days before diagnosis by farm staff (5). Additionally, cows diagnosed lame spent approximately 20 less minutes per day feeding 7 days prior to diagnosis (5). Healthy feedlot steers spent more time at the feed bunk than morbid steers (9). The use of radio frequency technology to obtain individual time spent at the feed bunk was able to detect morbid steers approximately 4 days earlier than trained feedlot personnel (10).

The objective of this study was to investigate the relationship between time spent feeding prepartum and periparturient health disorders, milk yield, milk composition, and milk somatic cell count (SCC) in Jersey cows using an initial dataset of 925 cows and with nulliparous cows (animals pregnant with their first calf) housed separately from primiparous and multiparous cows. Most previously published studies used smaller datasets, mixed parity groups, and Holstein cows.

## Materials and Methods

### Animals, Management, and Housing

The current study analyzed data collected during two previous experiments (11–14) conducted at a large commercial dairy farm (6,400 lactating dairy cows) in south–central Minnesota, USA. The study was carried out in accordance with the recommendations of the Institutional Animal Care and Use Committee (IACUC), and the protocol was approved by the committee. Consent was granted by the owners. The dataset combined cows from both studies as study was found not to be significant in the preliminary statistical analysis.

Prefresh (last 4 weeks of gestation) prepartum cows were housed in a 12-row low profile cross-ventilated barn and fed a totally mixed ration once daily with frequent feed push-ups to bring feed within reach of the cows at all times. Prepartum cows were fed from a feed alley by headlocks. All experimental pens had the same measurements of 31.7 m × 11.0 m and had 44 deep sand-bedded freestalls (229 cm L × 107 cm W) with a head-to-head configuration and 48 headlocks (each headlock measured 61 cm). Two water troughs were located in the pen and measured 366 cm × 56 cm; water was available at all times. One water trough was located at the end of the bank of freestalls, and a shared water trough was located between the treatment pen and an adjacent non-experimental pen.

Jersey cows were enrolled in the experiment 4 weeks prior to expected calving date, and all cows had a body condition score between 2 and 4 (1–5 scale; 1 = emaciated and 5 = obese) and locomotion score <3 (1–5 scale; 1 = normal and 5 = severely lame). Parity was based on lactation number at the time of enrollment and classified as nulliparous (cows pregnant with their first calf), primiparous (cows pregnant with their second calf), and multiparous (lactation ≥2). A total of 925 cows were used in the current study.

When cows demonstrated signs of calving, farm personnel moved the cows to an individual box stall. At day 1, post-calving cows were moved into a freestall pen with 240 stalls and 260 headlocks for 21 days, with a stocking rate not exceeding the number of stalls. Multiparous and primiparous cows were housed separately during the first 21 days after calving.

### Feeding Behavior Measurements

All enrolled cows were identified with a unique alphanumeric symbol on their backs using permanent hair dye either in black or blonde. Hair dye was applied from day 0 ± 1 relative to entering the treatment pens. To observe feeding behavior, each pen was equipped with three video cameras (Weldex, Cypress, CA, USA) connected to a digital video recording system (Channel Visions, Costa Mesa, CA, USA). Time spent feeding was measured using 10-min video scan sampling for 24-h periods (15). We chose to measure time spent feeding (also referred to as feeding time in the current study) as our feeding behavior observation because previous research indicated that time spent feeding was the most sensitive measure as it relates to prediction of health problems (5) and because of the feeding behavior methodology that could be logistically used in our study. For study 1, feeding behavior data were collected on days 1, 2, 3, and 7 of each week of the 4-week replicate, whereas for study 2, data were collected on days 2, 5, and 7 for week 1 of each replicate and days 2 and 5 for weeks 2–4. The goal was to have at least two 24-h periods of feeding behavior observation per cow per week. A cow was considered eating when the cow’s head was on the feed alley side of the headlocks. Video observation for the current study ceased when cows left the prepartum period treatment pens.

### Body Condition and Locomotion Score

At enrollment and on days 1 ± 1, 28 ± 3, and 56 ± 3 postpartum, all cows were scored for body condition [1 = emaciated to 5 = obese; (16)] and locomotion [1 = normal, 2 = imperfect locomotion, 3 = lame, 4 and 5 = severely lame; (17)].

### Blood Sampling and Analysis of Metabolites in Plasma

Blood samples were collected from all cows on days −18 ± 3, −11 ± 3, −4 ± 3, 3 ± 3, 10 ± 3, 17 ± 3, and 24 ± 3 relative to calving from the coccygeal vein or artery immediately after feeding while cows were restrained in self-locking headlocks. Samples were collected into evacuated tubes containing K2 EDTA (Becton Dickinson Vacutainer Systems, Franklin Lakes, NJ, USA). Tubes were placed in ice until centrifugation for plasma separation (1,200 × *g* for 15 min at 4°C). Plasma was aliquoted into microcentrifuge tubes and stored at −32°C until analysis.

Concentrations of beta-hydroxybutyrate (BHBA) were determined enzymatically [Ranbut, Randox Laboratories, Antrim, UK; (18)] from samples collected weekly from days in milk (DIM) 3 to 24.

### Clinical Examination and Definitions of Health Disorders

All cows were examined on DIM 1, 4 ± 1, 7 ± 1, 10 ± 1, and 13 ± 1 for the diagnosis of retained fetal membranes and metritis. Retained fetal membranes (RP) was defined as retention of a fetal membrane more than 24-h postpartum. Metritis was defined as cows with watery, pink or brown, and fetid uterine discharge. Acute metritis included the symptoms of metritis and presence of fever (>39.5°C) and anorexia. Cows were classified with subclinical ketosis (SCK) when BHBA concentrations were ≥1200 μmol/L. Clinical ketosis was not specifically recorded. All cows were observed once daily for displaced abomasum (DA) and thrice daily for mastitis. Cows were followed up to 14 DIM for mastitis and 60 DIM for DA. Cows considered healthy were not diagnosed with metritis, RP, SCK, DA, or mastitis up to 14 DIM and were not lame at DIM 1 or 35.

### Production Parameters

Cows were milked thrice daily. Milk yield, milk fat and protein contents, and SCC were recorded for individual cows during Dairy Herd Improvement Association (DHIA) monthly milk test. Energy-corrected milk yield was calculated for each cow using the formula ECM (kg) = [(kg of milk) × 0.327] + [(kg of fat) × 12.95] + [(kg of protein) × 7.2] (DHIA).

### Statistical Analysis

Data were analyzed using Proc Mixed of SAS (v 9.2 SAS Institute Inc., Cary, NC, USA). Cow was used as the experimental unit (*n* = 925). Preliminary statistical analysis determined no difference related to study, and data from the two studies were combined. Prepartum parity (nulliparous, primiparous, and multiparous) was tested independently after univariate analysis detected differences in time spent feeding (or feeding time) among parities. Daily feeding times were averaged for four prepartum periods, which were categorized by week prepartum: week −4 (day −28 to day −22), week −3 (day −21 to day −15), week −2 (day −14 to day −8), and week −1 (day −7 to day −1). Day of calving was excluded from analysis due to the cow leaving the treatment pen. A repeated statement included week and cow as the subject. The structure of covariance (compound, unstructured, or autoregressive) for the repeated statement was chosen according to the Bayesian Akaike information criteria. Fixed effects to the model included health status (disease event of interest vs. healthy), week relative to calving, and the interaction of health status by week relative to calving. Other covariates offered to the model included the pen temperature, pen stocking density, difference in body condition score from enrollment to day of calving, days housed in the prepartum pen, and other periparturient health events.

## Results

Table 1 shows the frequency and incidence of health events by parity. Combined parity incidence was 17.2% for metritis, 9.1% for acute metritis, 0.8% for DA, 7.5% for RP, 1.8% for mastitis, 2.5% for SCK, 1.4% for lameness at 1 DIM, and 3.9% for lameness at 35 DIM (Table 1). Some of the health disorders had a small number of animals represented in the dataset; therefore, these specific health disorders were not used for the final model analysis. An analysis comparing healthy cows with cows having either one or more than one health disorder was performed. In addition, we analyzed health disorders that had a representative number of animals per parity, namely metritis (metritis and acute metritis combined) and RP.

**Table 1:** **Health disorders (one, two, or more), displaced abomasum (DA), metritis, acute metritis, retained fetal membrane (RP), subclinical ketosis (SCK), mastitis up to 14 DIM, lame at DIM 1, and DIM 35 for nulliparous, primiparous, and multiparous Jersey cows**.

	Nulliparous^a^ (*n* = 316)		Primiparous^a^ (*n* = 318)		Multiparous^a^ (*n* = 291)		All cows (*n* = 925)	
	*n*	%	*n*	%	*n*	%	*n*	%
Healthy	209	66.1	223	70.1	185	63.6	617	66.7
One disorder	90	28.5	63	19.8	70	24.1	223	24.1
Two or more disorders	17	5.4	32	10.1	36	12.4	85	9.2
DA	0	0	2	0.6	4	1.7	6	0.6
Metritis	72	22.8	50	15.7	37	12.7	159	17.2
Acute metritis	43	13.5	21	6.6	20	6.9	84	9.1
RP	14	4.4	28	8.8	27	9.3	69	7.5
SCK	14	4.4	2	0.6	7	2.4	23	2.5
Mastitis	7	2.2	4	1.3	6	2.1	17	1.8
Lame 1 DIM	0	0	4	1.3	9	3.1	13	1.4
Lame 35 DIM	3	0.9	10	3.1	23	7.9	36	3.9

*^a^Parity classified at the time of enrollment when entering prepartum pens*.

### Healthy vs. Sick Cows

**Figure 1:**
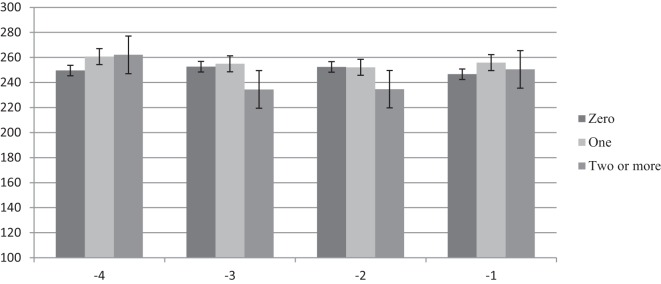
**Time spent feeding by week prepartum for nulliparous cows with zero, one, or two or more health events postpartum**.

**Figure 2:**
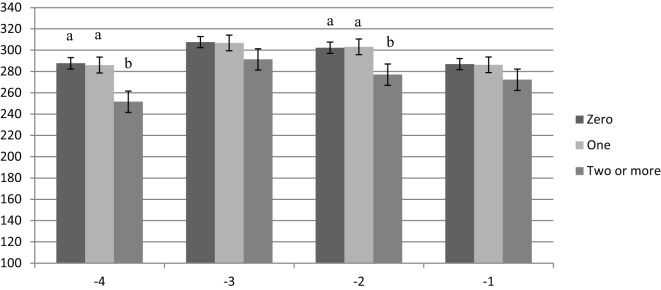
**Time spent feeding by week prepartum for primiparous cows with zero, one, or two or more health events postpartum**. a,b indicates difference between feeding time for cows with two or more health events vs. healthy or cows with only one health event within week prepartum (*P* < 0.05).

**Figure 3:**
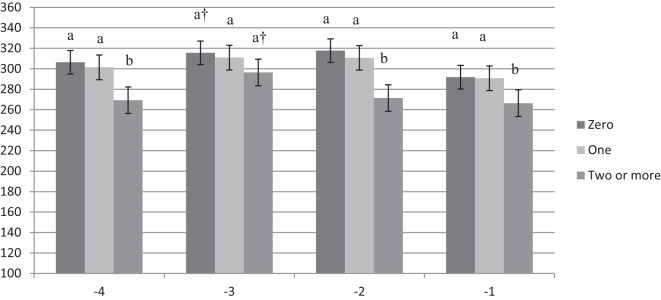
**Time spent feeding by week prepartum for multiparous cows with zero, one, or two or more health events postpartum**. a,b indicates difference between feeding time for cows with two or more health events vs. healthy or cows with only one health event within week prepartum (*P* < 0.05). † indicates a tendency between feeding time for cows with two or more health events vs. cows that did not have a health event (*P* = 0.08).

Results for this analysis are presented in Figures 1–3. There was no difference in feeding time for nulliparous cows that were diagnosed as healthy, with one health disorder, or with two or more health disorders (Figure 1). Primiparous cows were found to reduce their daily feeding time by 36 min or 12.5% in week −4 and by 25 min or 8.3% in week −2 compared with healthy primiparous cows (*P* < 0.05). Healthy cows and cows with only one health disorder had similar feeding times (Figure 2). Multiparous cows with two of more health disorders reduced their daily time spent feeding by approximately 32 min or 10.4% compared with healthy cows across all 4 weeks prepartum (week −1, 2, and 4, *P* < 0.05; week −3, *P* = 0.08). The greatest difference was in week −2, when they reduced their feeding time by 47 min or 14.8% as compared with healthy cows (*P* < 0.05). There was no difference in time spent feeding between healthy multiparous cows and multiparous cows with only one health disorder (Figure 3).

### Metritis

Incidence of metritis for nulliparous, primiparous, and multiparous animals were 22.8, 15.7, and 12.7%, respectively. This dataset includes cows that were diagnosed with acute metritis, and we did not conduct a separate analysis for acute metritis only as the number of primiparous and multiparous cows with this health disorder was less than 25. Nulliparous and primiparous cows with metritis did not have significant changes in prepartum feeding time versus healthy counterparts (Table 2). Multiparous cows diagnosed with metritis spent approximately 20 min or 6.5% less time feeding during week −3 and week −2 prepartum (*P* = 0.02 and *P* = 0.04, respectively) than healthy counterparts, however, did not differ from healthy cows in week −1 (*P* = 0.43) and week −4 (*P* = 0.51).

**Table 2:** **Associations between metritis and daily feeding time of Jersey cows from week −4 to week −1 according to parity (LSmean ± SE)**.

Parity	Week prepartum	Healthy (min/day)	Metritis (min/day)	Difference (min/day)	Health status (*P*-value)
**Nulliparous^a^**					
	−4	254 ± 10	267 ± 11	13 ± 7	0.17
	−3	256 ± 10	260 ± 11	4 ± 9	0.60
	−2	257 ± 10	253 ± 10	−5 ± 9	0.64
	−1	257 ± 10	268 ± 10	11 ± 10	0.085
**Primiparous^a^**					
	−4	277 ± 10	279 ± 12	2 ± 12	0.86
	−3	298 ± 9	298 ± 9	0 ± 11	0.68
	−2	292 ± 8	294 ± 8	2 ± 11	0.83
	−1	276 ± 9	280 ± 9	5 ± 11	0.68
**Multiparous^a^**					
	−4	307 ± 6	298 ± 12	−9 ± 12	0.51
	−3	309 ± 4	289 ± 9	−20 ± 10	0.02
	−2	308 ± 3	289 ± 8	−20 ± 9	0.04
	−1	293 ± 4	287 ± 8	−6 ± 9	0.43

*^a^Parity classified at the time of enrollment in prepartum pen*.

### Retained Fetal Membranes

**Figure 4:**
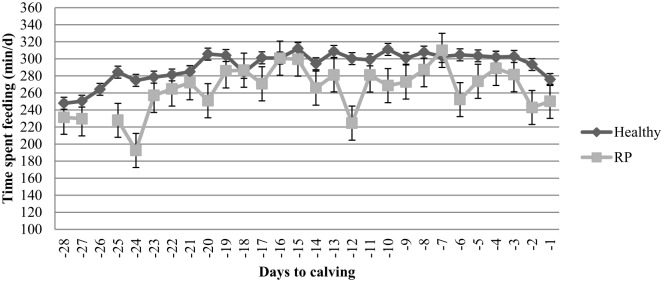
**Prepartum feeding time of healthy primiparous cows and primiparous cows diagnosed with retained fetal membrane (RP) post calving**. Primiparous cows diagnosed with a RP spent 28 ± 10 fewer minutes per day feeding than healthy primiparous cows (*P* = 0.005) up to 4 weeks prior to calving.

The incidence of RP was 4.4% for nulliparous, 8.8% for primiparous, and 9.3% for multiparous cows. Nulliparous and multiparous with RP did not differ in feeding times as compared with healthy nulliparous and multiparous cows, respectively (Table 3). Overall primiparous cows with RP spent 28 ± 10 fewer minutes per day feeding than healthy primiparous cows (*P* = 0.005; Figure 4). Primiparous cows with RP reduced their feeding time during weeks −4, −2, and −1 by 12, 11, and 9%, respectively. There was a numeric percentage decrease of 6% during week −3 for primiparous cows with a RP (*P* = 0.13).

**Table 3:** **Associations between retained fetal membrane (RP) and daily feeding time of Jersey dairy cows by parity from week −4 to week −1 before calving according to parity (LSmean ± SE)**.

Parity	Week prepartum	Average feeding time (min/day)			Health status *P*-value
		Healthy	RP	Difference	
**Nulliparous^a^**					
	−4	249 ± 4	233 ± 20	−15 ± 21	0.46
	−3	250 ± 4	241 ± 16	−9 ± 16	0.58
	−2	253 ± 4	223 ± 16	−30 ± 16	0.07
	−1	249 ± 4	254 ± 16	5 ± 17	0.78
**Primiparous^a^**					
	−4	269 ± 4	237 ± 15	−32 ± 16	0.047
	−3	299 ± 4	281 ± 11	−18 ± 12	0.13
	−2	303 ± 4	269 ± 11	−34 ± 11	0.003
	−1	297 ± 4	270 ± 11	−27 ± 11	0.016
**Multiparous^a^**					
	−4	286 ± 7	273 ± 17	−12 ± 16	0.45
	−3	296 ± 5	302 ± 14	5 ± 14	0.70
	−2	300 ± 4	289 ± 12	−11 ± 13	0.38
	−1	273 ± 6	269 ± 12	−5 ± 13	0.71

*^a^Parity classified at the time of enrollment in prepartum pen*.

### Milk Yield and Composition

There were no associations between time spent feeding prepartum and milk yield (*P* = 0.43), energy-corrected milk yield (*P* = 0.68), fat-corrected milk yield (*P* = 0.42), milk protein yield (*P* = 0.13), milk protein % (*P* = 0.19), milk fat yield (*P* = 0.80), milk fat % (*P* = 0.20), or SCC (*P* = 0.11).

## Discussion

Our results provide some evidence for using prepartum feeding behavior data to identify primiparous and multiparous Jersey cows at risk for health disorders early after calving with limited sampling (2–4 days per week). A reduction in time spent feeding prepartum was associated with having more than one health disorder during the periparturient period for primiparous and multiparous cows. Because we had a representative number of animals with certain specific health disorders, we also found that reduction in feeding time was associated with metritis and RP. To our knowledge, this study was the first to show an association between feeding time and RP. Previous research has detected associations between feeding time of dairy cows and metritis (7, 8, 19), clinical ketosis (20), and lameness (5, 21, 22).

In the current study, nulliparous cows were housed separately from primiparous and multiparous cows, and this allowed us to evaluate their behavior more specifically than some previous studies. Parity was examined separately due to the differences in feeding times that existed among parities. Nulliparous cows spent fewer minutes per day feeding overall than both primiparous and multiparous cows (approximately 40 ± 4 and 29 ± 4 min/day, respectively). We housed nulliparous cows separately from the older animals to allow for the characterization of their behavior without hindrance from competition. Prepartum nulliparous cows typically have lower DMI per day than multiparous cows (23), and shorter feeding times could be considered a proxy for DMI.

Although primiparous and multiparous cows were housed together, preliminary analysis showed differences in feeding time between the two groups and, therefore, they were not combined in the mixed models. Huzzey et al. (8) reported that primiparous Holstein cows had a slower feeding rate than multiparous cows. In the current study, primiparous Jersey animals spent 12 ± 4 min more time feeding than multiparous cows.

Overall disease incidence in the current study was lower than some previously reported research results. However, our initial dataset included a total of 925 cows with a representative number of cows with health disorders (*n* = 308) available for analysis as compared with previous studies. Patbandha et al. (19) reported a 40% incidence of metritis in crossbred cows, and Urton et al. (7) reported 69% incidence in Holstein cows and heifers. In a small study with 22 first lactation Jersey cows (24), only two cases (9%) of ketosis and six cases (27%) of mastitis were reported, whereas no cases of DA or metritis were found. Typically, periparturient health disorders are interrelated rather than one single health event. LeBlanc et al. (25) indicated that RP, metritis, and increased concentrations of BHBA were associated with an increased risk for DA. Therefore, the analysis of combined health disorders could be a better predictor for cows at high risk for disease, culling, or mortality.

Nulliparous cows had the greatest incidence of metritis (28%) in the current study compared with primiparous and multiparous cows, although the incidence in the current study was lower than the 49% reported in Holsteins by Giuliodori et al. (26). This is most likely due to the reduced calving difficulty in the Jersey breed (27) and reducing the trauma to the uterine wall from assisted calvings. We did not find an association between time spent feeding prepartum and metritis in spite of the fact that nulliparous cows with metritis were a representative number in our dataset with 72 sick animals included in the analysis. This could be an indication that younger animals are more resilient and able to cope better than older animals. However, it is unknown whether they altered other feeding characteristics, such as dry matter intake or feeding rate.

Huzzey et al. (8) found that the DM intake of severely metritic cows was depressed 2 weeks prior to calving, and those cows continued to consume less feed 3 weeks after calving. A decrease in feeding time was observed 2 weeks prior to the diagnosis of clinical metritis. Patbandha et al. (28) recorded prepartum feeding time of 20 multiparous Holstein–Friesian crossbred cows and reported that cows with daily feeding time of 284.5 min/day during the period day −6 to day −2 were more likely to develop metritis (Se = 75% and Sp = 91.7%) compared with cows above that threshold. Those metritic cows had lower number of feeding bouts and higher inactive standing time compared with normal cows. For cows diagnosed with metritis in our study, 25.9% also had RP, 1.8% DA, 5.9% SCK, 1.8% lameness, and 2.3% a mastitis case by 14 DIM.

Previous studies have cited RP and dystocia as risk factors for metritis (8, 26, 29). Additional risk factors for metritis have included breed, parity, calving season, ketosis, milk fever, and mastitis during the dry period (29), stillborn birth and elevated NEFA concentrations prepartum (26). Retained placenta has been linked to immune suppression and elevated NEFA concentrations with ketosis being a risk factor for RP (30). Cows with RP were shown to have significantly lower neutrophil function before calving and up to 2 weeks postpartum (31). Elevated lipid mobilization increases the risk for fatty liver, reduction of DMI, and disease events (25). To our knowledge, this is the first study to find an association between prepartum feeding time and RP.

Our objective was to investigate whether cows in the prepartum period would differ in feeding behavior from their herd average if they became sick during the postpartum period. If they differed, these cows could be flagged before or at calving as “more at risk” so the dairy producer could take action and possibly provide a more comfortable environment, more supportive preventive options such as calcium, probiotics, electrolytes, or other options that might help those animals transition to the new lactation more successfully. Our results indicate that nulliparous animals might have less of a change in behavior than older cows, potentially making it more difficult to use feeding behavior as a tool to identify those younger animals at risk. However, older cows with more than one health disorder during the periparturient period reduced their time spent feeding compared with healthy cows or cows with only one health disorder, therefore, suggesting that prepartum feeding behavior can be a predictor of older cows at most risk. Flagging these animals and providing them with more observation and care could reduce postpartum mortality and culling and improve animal welfare and dairy farm profitability. We caution that these results might not necessarily be applicable to all breeds of dairy cattle as our study used only Jersey cows.

Some of the limitations of our study also include not measuring feeding behavior throughout the entire prepartum period and the immediate postpartum period and only using time spent feeding as our behavior measurement. More research is needed to investigate the use of real-time monitoring systems that could automate the measurement of individual cow feeding behavior and collect more data points for each animal, including time spent feeding, number of feeding bouts, and feeding bout duration. In addition, especially with the availability of such technologies, more research is needed to determine whether deviations from each animal’s own daily feeding behavior, may be still compared with their specific cohorts, would be a more accurate measurement.

## Conclusion

Our results provide evidence that monitoring time spent feeding prepartum in Jersey cows could aid in the identification of cows at higher risk of a periparturient health disorder, especially in older cows. Prepartum multiparous cows (lactation ≥2) with two or more health disorders decreased their prepartum feeding time compared with healthy counterparts more than nulliparous animals did. In addition, results indicate that feeding behavior needs to be evaluated within specific parity cohorts and not comparing the behavior of a cow to the entire herd.

## Author Contributions

KL – Ph.D. graduate student who collected and summarized behavior data, conducted statistical analysis, and wrote manuscript draft. PS – Ph.D. student who collected and summarized health data and helped to edit manuscript. RC – developed health protocols. PS – advisor and helped to edit manuscript. ME – corresponding author and developed behavior protocols. KL – advisor. PS – co-advisor, edited the manuscript draft, and coordinated the study.

## Conflict of Interest Statement

The authors declare that the research was conducted in the absence of any commercial or financial relationships that could be construed as a potential conflict of interest.
